# Influence of Spatial Dispersion on Propagation Properties of Waveguides Based on Hyperbolic Metamaterial

**DOI:** 10.3390/ma14226885

**Published:** 2021-11-15

**Authors:** Bartosz Janaszek, Anna Tyszka-Zawadzka, Paweł Szczepański

**Affiliations:** 1 Institute of Microelectronics and Optoelectronics, Warsaw University of Technology, Koszykowa 75, 00-665 Warsaw, Poland; anna.zawadzka1@pw.edu.pl (A.T.-Z.); pawel.szczepanski@pw.edu.pl (P.S.); 2 National Institute of Telecommunications, 1 Szachowa, 04-894 Warsaw, Poland

**Keywords:** spatial dispersion, waveguides, propagation properties, hyperbolic metamaterials

## Abstract

In this work, we study the effect of spatial dispersion on propagation properties of planar waveguides with the core layer formed by hyperbolic metamaterial (HMM). In our case, the influence of spatial dispersion was controlled by changing the unit cell’s dimensions. Our analysis revealed a number of new effects arising in the considered waveguides, which cannot be predicted with the help of local approximation, including mode degeneration (existence of additional branch of TE and TM *high-β* modes), power flow inversion, propagation gap, and plasmonic-like modes characterized with long distance propagation. Additionally, for the first time we reported unusual characteristic points appearing for the *high-β* TM mode of each order corresponding to a single waveguide width for which power flow tends to zero and mode stopping occurs.

## 1. Introduction

By utilizing nanostructurization at the subwavelength scale, optical metamaterials provide a means for controlling light propagation that is not available in conventional media [1,2,3,4]. In the last decade, uniaxial anisotropic metamaterial possessing hyperbolic dispersion, called hyperbolic metamaterials (HMMs), have attracted special interest due to their relatively simple technological realization and unique optical properties. In particular, this type of metamaterial can be utilized in sub-diffraction imaging [5], electromagnetic cloaking [6,7], photonic-density of states manipulation [8], spontaneous emission engineering [9,10], and biosensing [11,12,13]. More recently, HMMs have been recognized as a very prospective building block for waveguiding systems, allowing a number of unique properties to be obtained, such as simultaneous propagation of plasmons and bulk waves [13,14], the coexistence of forward and backward modes (i.e., two modes of the same direction of phase velocity and the contrary signs of power flows) [15], light enhancement [16], and light slowing or stopping [17,18].

Typically, hyperbolic metamaterials are effectively described via the use of the local effective medium theory (local EMT), as uniaxially anisotropic media with permittivity tensors having components of opposite signs [19,20]. Such an approach provides good agreement between theory and experiment when characteristic dimensions of described photonic nanostructures are much smaller than the considered wavelength [21,22]. However, more recent studies reported substantial deviations between the electromagnetic response predicted with the help of the local EMT and the actual behavior of realistic nanostructures [23,24,25,26]. The origin of these discrepancies has been identified as the influence of spatial dispersion, which can be described as the wavevector-dependence of permittivity, which may cause the occurrence of an additional optical axis [27]. Until now, it has been proven that spatial dispersion (i.e., nonlocality) may not only be considered as a factor deteriorating the intended performance [28,29] but also can be exploited to unveil new effects that cannot be predicted with local approximation [26,30]. Especially, nonlocality may be used to enhance spontaneous emission [31] and nonlinear optical response in metamaterial nanostructures [32]. It has also been shown that spatial dispersion may strongly affect plasmonic mode propagation in metamaterials based on nanowires [24,33].

More recently, an increasing number of studies has been devoted to new effects that arise in HMMs in the presence of spatial dispersion, including nonlocal quantum gain of plasmons [34], inverse transition radiation of controllable direction [35], large enhancement of decay rate of an emitter located inside a hyperbolic metamaterial [36], and blueshift of intramolecular charge transfer emission [37]. It has also been shown that with the help of nonlocality, it is possible to shape effective dispersion of HMMs [38] as well as to obtain highly selective spatial filtering [39] or nonmagnetic optical isolation [40,41]. So far, the studies dedicated to spatial dispersion in hyperbolic metamaterials have covered mainly phenomena in bulk HMM structures. On the other hand, the influence of spatial dispersion on waveguiding properties has been rarely addressed in the existing state of the art. Until now, it has been considered only in terms of nonlocal bulk plasma mode propagation (Langmuir modes) [42]. In particular, it has been shown that spatial dispersion in waveguides leads to a break of the singularity in the density of optical states and a suppression of negative dispersion induced by anisotropy [42]. Thus, the nonlocal propagation properties of waveguiding systems based on hyperbolic metamaterials is still an unexplored and promising scientific area.

In this paper, for the first time, we investigate the influence of spatial dispersion on propagation properties of guided modes in the hyperbolic metamaterial waveguide. The considered system is a symmetric planar waveguide composed of HMM core and air cladding. By appropriate design of the HMM structure, i.e., modifying the size of the unit cell, strong spatial dispersion can be obtained and employed to unveil unique propagation properties, which cannot be predicted with the help of the local approach. In the course of the analysis, we demonstrated a number of new effects arising in the presence of spatial dispersion, including mode degeneracy leading to occurrence of an additional branch of TE/TM modes characterized with a high propagation constant and high modal confinement (high-β modes). Moreover, it has been shown that high-β TM modes may reveal a characteristic point corresponding to a waveguide width for which the direction of power flow is reversed with respect to the mode propagation. In particular, this phenomenon may be considered as a new mechanism for stopping propagation of a selected TM mode. Thus, the presented results revealed that spatial dispersion substantially influences propagation properties of waveguides based on HMM and may lead to new effects that cannot be predicted with the help of local approximation.

## 2. Theory

**Figure 1:**
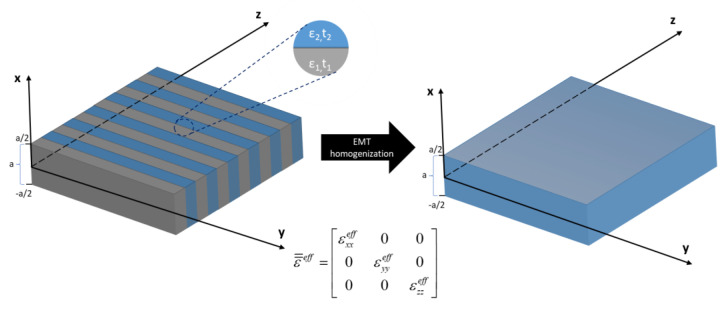
Schematic representation of HMM waveguide core layer homogenization.

In this section, we present a theoretical model for analysis of propagation in hyperbolic metamaterial waveguides. The considered system, shown in Figure 1, is a symmetrical planar waveguide composed of an HMM core and air cladding ($\varepsilon_{c}$ = 1). The HMM core of the width ”a” consists of subsequent ultrathin layers of two different materials with relative electric permittivities $\varepsilon_{\left(1,2\right)}$ and layer thicknesses $t_{\left(1,2\right)}$ (see Figure 1).

We assume the direction of propagation to be along the *z*-axis. The HMM medium forming the core layer can be described as an anisotropic medium with an effective permittivity tensor of the following form [19]:

(1) $${\bar{\bar{\varepsilon }}}^{eff}=\left[\begin{array}{ccc} \varepsilon_{xx}^{eff} & 0 & 0\\ 0 & \varepsilon_{yy}^{eff} & 0\\ 0 & 0 & \varepsilon_{zz}^{eff} \end{array}\right]$$

where $\varepsilon_{xx}^{eff}$, $\varepsilon_{yy}^{eff}$, $\varepsilon_{zz}^{eff}\;$ are the effective permittivity components determined with use of the effective medium theory [21].

In such a medium, the dispersion relations for transverse electric (TE) and transverse magnetic (TM) polarized waves propagating in the x–z plane may be represented with

(2) $$k_{x}^{2}+k_{z}^{2}=\varepsilon_{yy}^{eff}k_{0}^{2}\;(\mathrm{TE})$$

and

(3) $$\frac{k_{x}^{2}}{\varepsilon_{zz}^{eff}}+\frac{k_{z}^{2}}{\varepsilon_{xx}^{eff}}=k_{0}^{2},\;(\mathrm{TM}),$$

respectively. Here, $k_{0}$ is the freespace wavevector, while $k_{x}$, $k_{z}$ are related to components of the wavevector inside the medium.

In order to describe propagation properties of the waveguide shown in Figure 1, we solve Maxwell’s equations with appropriate boundary conditions for a nonmagnetic waveguide system described with a permittivity tensor in the following form:

(4) $$\overset{=}{\varepsilon }=\left\{ \begin{array}{l} \begin{array}{cc} \varepsilon_{c}\cdot \overset{=}{I}, & x<-a/2\\ {\overset{=}{\varepsilon }}_{}^{eff}, & -a/2<x<a/2 \end{array}\\ \begin{array}{cc} \varepsilon_{c}\cdot \overset{=}{I}, & \end{array}x>a/2 \end{array}\right.$$

where $\overset{=}{I}$ is a unit matrix.

By assuming continuity of electric and magnetic fields at interfaces *x* = a/2 and *x* = −a/2, we determine the characteristic eigenvalue equations for TE and TM oscillatory modes: for TE modes

(5) $$\tan\left(\gamma_{f}^{TE}\frac{a}{2}\right)=\frac{\gamma_{c}}{\gamma_{f}^{TE}}\frac{\mu_{o}}{\mu_{c}},\;\mathrm{for}\;\mathrm{even}\;\mathrm{modes}$$

(6) $$\cot\left(\gamma_{f}^{TE}\frac{a}{2}\right)=-\frac{\gamma_{c}}{\gamma_{f}^{TE}}\frac{\mu_{o}}{\mu_{c}},\;\mathrm{for}\;\mathrm{odd}\;\mathrm{modes}\;$$

for TM modes

(7) $$\tan\left(\gamma_{f}^{TM}\frac{a}{2}\right)=\frac{\gamma_{c}}{\gamma_{f}^{TM}}\frac{\varepsilon_{zz}^{eff}}{\varepsilon_{c}},\;\mathrm{for}\;\mathrm{even}\;\mathrm{modes}$$

(8) $$\cot\left(\gamma_{f}^{TM}\frac{a}{2}\right)=-\frac{\gamma_{c}}{\gamma_{f}^{TM}}\frac{\varepsilon_{zz}^{eff}}{\varepsilon_{c}},\;\mathrm{for}\;\mathrm{odd}\;\mathrm{modes}$$

where $\gamma_{f}^{TE}=\sqrt{k_{o}^{2}\varepsilon_{yy}^{eff}-\beta^{2}}$, $\gamma_{f}^{TM}=\sqrt{k_{o}^{2}\varepsilon_{zz}^{eff}-\beta^{2}\frac{\varepsilon_{zz}^{eff}}{\varepsilon_{xx}^{eff}}}$, $\gamma_{c}=\sqrt{\beta^{2}-k_{o}^{2}\varepsilon_{c}}$ and β is the complex propagation constant of the waveguide mode obtained by solving the given eigenmode equation. The real part of the propagation constant β is related to phase velocity, while the imaginary part determines the propagation length, $L_{propagation}=\frac{2}{Im\left(\beta \right)}$, of the waveguide mode [43]. It is noteworthy that Equations (5)–(8) can be applied for any nonmagnetic symmetric waveguide with anisotropic medium acting as a core layer. Moreover, via use of the electric field distribution, it is possible to calculate power flow $P_{flow}=\frac{\int S_{z}dx}{\int \left|S_{z}\right|dx}$, where Sz=12Re(E→×H*→)z and $\vec{E}$, $\vec{H}$ are electric and magnetic field vectors of the given mode.

In further analysis, we employ Equations (5)–(8) to calculate propagation constants of TE and TM modes for different waveguide widths. To illustrate the difference between local and nonlocal propagation properties, we employ two different approaches for describing the HMM core layer, namely local and nonlocal effective medium theory (EMT).

In the case of the local EMT approach, the HMM core layer can be described as a homogeneous uniaxial anisotropic medium with the effective diagonal permittivity tensor having components of the following forms [19]:

(9) $$\varepsilon_{xx,yy}^{eff}=\varepsilon_{xx,yy}^{loc}=\frac{t_{1}\varepsilon_{1}(\omega )+t_{1}\varepsilon_{1}(\omega )}{t_{1}+t_{2}}$$

(10) $$\varepsilon_{zz}^{eff}=\varepsilon_{zz}^{loc}=\frac{\varepsilon_{1}(\omega )\varepsilon_{2}(\omega )(t_{1}+t_{2})}{t_{1}\varepsilon_{2}(\omega )+t_{2}\varepsilon_{1}(\omega )}$$

However, the local approximation is only valid for wavelengths much longer than the dimension of the unit cell, i.e., t/λ→0, where $t=t_{1}+t_{2}$, and when the spatial dispersion is negligible [21].

To predict the influence of the spatial dispersion on the waveguiding properties in the considered system, we need to employ a more rigorous method, which accounts for the wavevector-dependence of the permittivity tensor components. For this aim, similarly as in our previous research [38,39], we employ the formalism proposed by Chern [27], which states that a two-constituent multilayer medium can be effectively described with a biaxial anisotropic medium characterized with a diagonal permittivity tensor $\overset{¯}{\overset{¯}{\varepsilon }}\left(\omega ,\vec{k}\right)=diag\left(\varepsilon_{xx}^{nloc},\varepsilon_{yy}^{nloc},\varepsilon_{zz}^{nloc}\right)$, having components of the following form:

(11) $$\varepsilon_{xx}^{eff}=\varepsilon_{xx}^{nloc}=\frac{\varepsilon_{xx}^{loc}-\frac{\alpha }{12}k_{0}^{2}t^{2}}{1-\frac{1}{12}k_{z}^{2}t^{2}}$$

(12) $$\varepsilon_{yy}^{eff}=\varepsilon_{yy}^{nloc}=\varepsilon_{yy}^{loc}\left(1+\frac{1}{6}k_{0}^{2}t^{2}\right)+\frac{t^{2}}{12k_{0}^{2}}\left(k_{z}^{4}-k_{x}^{4}\right)-\frac{\alpha }{12}k_{0}^{2}t^{2}$$

(13) $$\varepsilon_{zz}^{eff}=\varepsilon_{zz}^{nloc}=\frac{\varepsilon_{zz}^{loc}-\frac{\alpha }{12}k_{0}^{2}t^{2}}{1+\frac{\varepsilon_{zz}^{loc}}{\varepsilon_{xx}^{loc}}\left(\frac{\rho }{12}k_{x}^{2}t^{2}-\frac{\gamma }{6}k_{0}^{2}t^{2}\right)}$$

where *α*, *β*, *γ* are as follows:

(14) $$\alpha =\left[f_{1}^{2}\varepsilon_{1}+\left(1-f_{1}^{2}\right)\varepsilon_{2}\right]\left[\left(1-f_{2}^{2}\right)\varepsilon_{1}+f_{2}^{2}\varepsilon_{2}\right]$$

(15) $$\rho =\frac{1}{\varepsilon_{1}\varepsilon_{2}}\left[\left(1-2f_{1}f_{2}\right)\varepsilon_{1}+2f_{1}f_{2}\varepsilon_{2}\right]\left[2f_{1}f_{2}\varepsilon_{1}+\left(1-2f_{1}f_{2}\right)\varepsilon_{2}\right]$$

(16) $$\gamma =\frac{1}{\varepsilon_{1}\varepsilon_{2}}\left[f_{1}^{3}f_{2}\varepsilon_{1}^{3}+f_{1}\left(1-2f_{1}^{2}f_{2}+f_{2}^{3}\right)\varepsilon_{1}^{2}\varepsilon_{2}+f_{2}\left(1-2f_{2}^{2}f_{1}+f_{1}^{3}\right)\varepsilon_{1}\varepsilon_{2}^{2}+f_{1}f_{2}^{3}\varepsilon_{2}^{3}\right]$$

and $f_{1,2}=t_{1,2}/t$ are the filling factors, $k_{0}=2\pi /\lambda$ is the free space wavevector, and $k_{x}$, $k_{z}$ are components of wavevector of the wave inside the medium.

In order to obtain dispersion characteristics for TE and TM modes propagating in an HMM waveguide, we solve the characteristic equations (Equations (5)–(8)) for the core layer described with both local and nonlocal EMT. It is worth noting that in the case of nonlocal description, the effective permittivity of the waveguide core depends on the value of the propagation constant of the given mode. Thus, the nonlocal permittivity and solution of the characteristic equations must be self-consistent.

## 3. Results and Discussion

Within the scope of our analysis, we considered a symmetric HMM waveguide cladded with air ($\varepsilon_{air}\approx 1$) (see Figure 1). We assumed that the unit cell of the HMM is composed of a monolayer graphene, i.e., $\varepsilon_{1}=\varepsilon_{graphene}$, $t_{1}=t_{graphene}=0.35\;\mathrm{nm}$, acting as a plasmonic material characterized with permittivity described with the Kubo formula [44], and a dielectric layer formed with niobium pentoxide (Nb_2_O_5_) $\varepsilon_{2}=\varepsilon_{Nb2O5}$, which can be described with the Tauc–Lorentz model [45]. It is worth underlining that the employed materials are considered as local, and all observed nonlocal effects arise from periodical arrangements of the HMM structure [27,38]. Since the presented results originate from nonlocality rather than material properties, the selection of these particular materials does not limit the generality of the presented analysis.

### 3.1. The Local and Nonlocal Response of the Guiding HMM Medium

**Figure 2:**
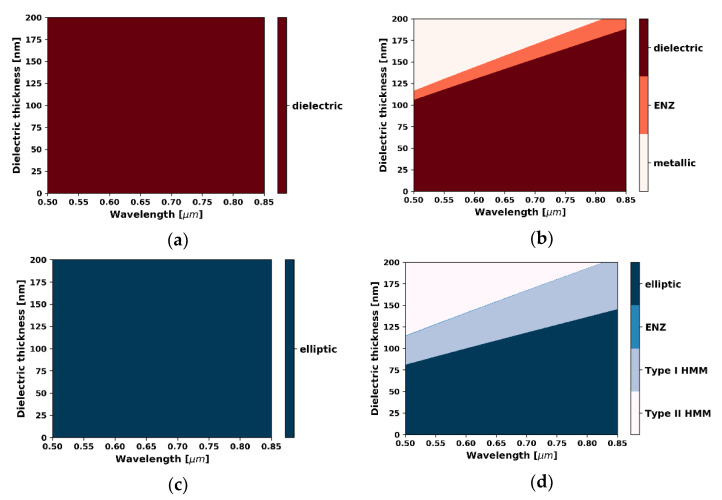
Maps of effective dispersion types of the HMM medium described with local (**a**,**c**) and nonlocal EMT (**b**,**d**) plotted as a function of wavelength and dielectric layer thickness for TE- (**a**,**b**) and TM-polarized waves (**c**,**d**) and for $\vec{k}=\left[\begin{array}{ccc} k_{x}, & k_{y}, & k_{z} \end{array}\right]=\left[\begin{array}{ccc} 0, & 0, & k_{0} \end{array}\right]$.

Thus, the HMM core layer is described as uniform uniaxial or biaxial anisotropic medium with the use of local or nonlocal EMT (see Equations (9)–(16), respectively), where $\varepsilon_{1}=\varepsilon_{graphene}$, $t_{1}=t_{graphene}=0.35\;\mathrm{nm}$ and $\varepsilon_{2}=\varepsilon_{Nb2O5}$, $t_{2}=t_{Nb2O5}$. Firstly, we investigated the difference between local and nonlocal responses of HMM medium forming the core layer. In Figure 2a–d, the characteristics illustrating occurrence of dispersion types for TE- and TM-polarized waves, plotted versus wavelengths and various thicknesses of the dielectric layer in the unit cell, were calculated via use of local and nonlocal EMT; see Equations (9)–(13) with $\vec{k}=\left[\begin{array}{ccc} k_{x}, & k_{y}, & k_{z} \end{array}\right]=\left[\begin{array}{ccc} 0, & 0, & k_{0} \end{array}\right]$.

Along with our previous research [38,39,40], we considered changing the dimensions of the unit cell, in this case dielectric layer thickness, as a means for controlling the influence of nonlocality on the effective dispersion of an anisotropic metamaterial. It is worth underlining that the range of considered thicknesses is in line with the applicability of the nonlocal EMT approach, i.e., $t<\lambda_{0}$ [27]. Within the considered spectral range, nonlocal EMT, in contrast to the local approach, predicts that by changing dielectric thickness it is possible to alter the dispersion type of the HMM core layer (compare Figure 2a,b). Moreover, for a given wavelength, the type of dispersion can be freely adjusted by increasing the dielectric layer thickness, as seen in Figure 2b,d. In particular, it is possible to switch from dielectric ($\varepsilon_{yy}^{eff}>0$) to metallic ($\varepsilon_{yy}^{eff}<0$) dispersion for TE waves and from elliptic ($\varepsilon_{xx}^{eff}>0,\varepsilon_{zz}^{eff}>0$) to type II hyperbolic ($\varepsilon_{xx}^{eff}<0,\varepsilon_{zz}^{eff}>0$) dispersion for TM waves. Since the spatial dispersion is a result of interactions between plasmons propagating at each metal/dielectric interface, changing the separation distance may lead to such substantial alterations of effective dispersion [38,43]. It is worth underlining that the nonlocal EMT approach predicts influence of wavevector components on effective permittivity of the guiding medium. This property is of key importance in the analysis of propagation in metamaterial waveguides for which the propagation constant may achieve extreme values. Since TE and TM modes of waveguide have different electric field components, i.e., $E_{y}$ and $E_{x},E_{z}$, respectively, they perceive types of dispersion based on various permittivity components, i.e., $\varepsilon_{yy}^{eff}$ for TE and $\varepsilon_{xx}^{eff},\varepsilon_{zz}^{eff}$ for TM modes; see Equations (5)–(8). Thus, it can be expected that, under certain conditions, modes of different types (TE/TM) and propagation constants may perceive various types of dispersion of the guiding medium.

### 3.2. Analysis of Propagation Properties

In our analysis, we considered four waveguides with different HMM guiding media composed of unit cells with various thicknesses of dielectric layers, i.e., $t_{Nb2O5}$ = 5, 80, 110, and 175 nm. According to nonlocal EMT, each selected dielectric thickness corresponds to different dispersion type of the core layer for the assumed wavelength λ=0.55 μm (see Figure 2b,d). In our analysis, we considered the propagation constant and propagation length for TE and TM waveguide modes of subsequent orders m = 1, 2, 3, 4. Moreover, for each mode, the direction of power flow was calculated and denoted with left- and right-oriented arrows, referring to antiparallel and parallel with respect to propagation direction of the mode. To determine the influence of nonlocality, we compared the propagation properties of a waveguide with the HMM core layer described with the local and nonlocal EMT approach.

#### 3.2.1. Case 1—The Core Layer with 5 nm Dielectric Layer

**Figure 3:**
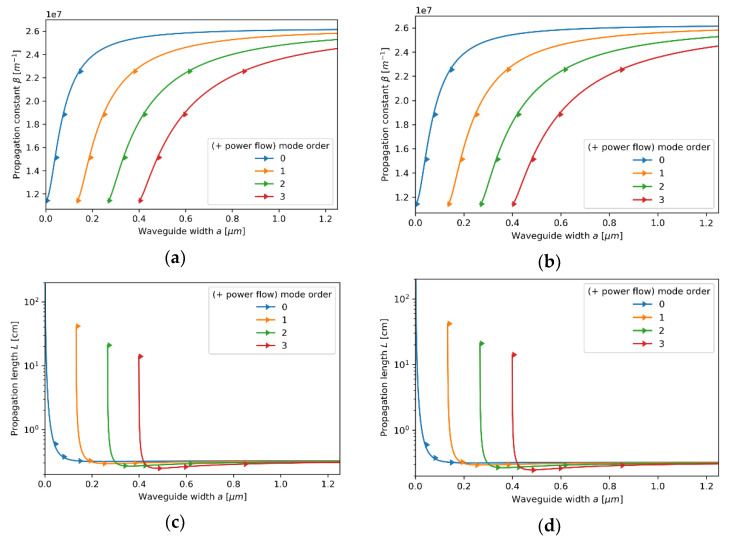
Propagation constant (**a**,**b**) and propagation length (**c**,**d**) of TE modes plotted versus waveguide width for an HMM waveguide with Nb2O5 layer of *t*_Nb2O5_ = 5 nm thickness embedded in the unit cell and described with local (**a**,**c**) and nonlocal EMT (**b**,**d**).

**Figure 4:**
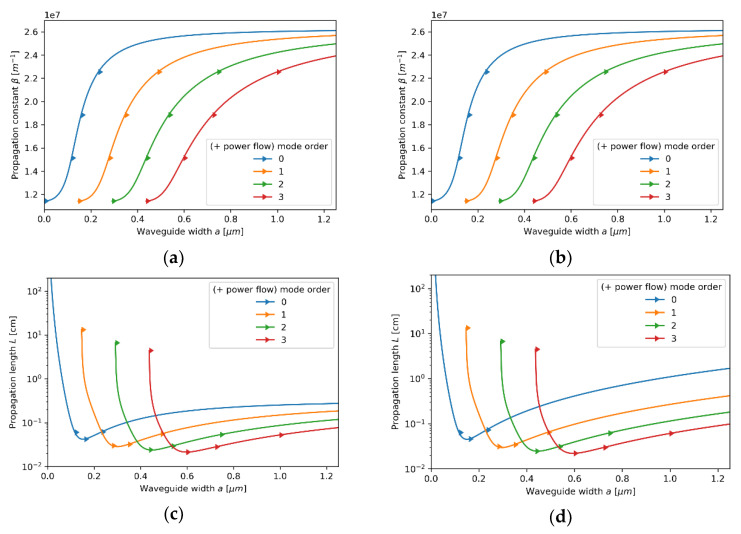
Propagation constant (**a**,**b**) and propagation length (**c**,**d**) of TM modes plotted versus waveguide width for an HMM waveguide with Nb2O5 layer of *t*_Nb2O5_ = 5 nm thickness embedded in the unit cell and described with local (**a**,**c**) and nonlocal EMT (**b**,**d**).

Let us start our analysis from the first case, i.e., the core layer with the unit cell composed of a monolayer graphene and a 5 nm Nb_2_O_5_ layer. Since the characteristic dimensions, i.e., t=5.35 nm, are much smaller than the wavelength considered, the locality condition t/λ→0 is satisfied, and thus the influence of nonlocality may be considered as almost negligible. In this case, we obtain excellent agreement between local and nonlocal propagation properties for both TE and TM waveguide modes; compare Figure 3a–d and Figure 4a–d. However, by accounting for the nonlocality, it is possible to obtain a small correction in propagation properties. Nonlocal description, in contrast to the local approximation, enables us to predict longer propagation length of TM modes that occurs for waveguide widths larger than a>0.6μm (compare Figure 4c,d). Moreover, both local and nonlocal approaches predict that for each mode, the power flow coincides with the propagation direction “*+z*” (Poynting vector is parallel to the direction of propagation), which is denoted in Figure 3a–d and Figure 4a–d with right-oriented arrows.

**Figure 5:**
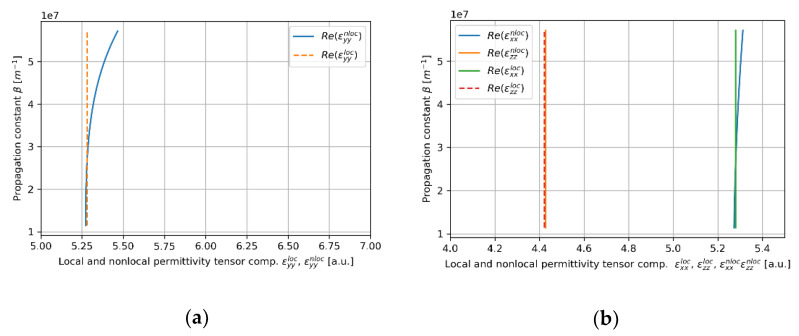
Real parts of components of effective local and nonlocal tensors (horizontal axis) of the core layer with dielectric layer thickness *t*_Nb2O5_ = 5 nm plotted versus propagation constant (vertical axis), which are encountered by TE (**a**) and TM waveguide modes (**b**).

In this case, the convergency of the local and nonlocal descriptions can be also observed in Figure 5a,b, where real parts of effective permittivity tensor components (horizontal axis) of the HMM core calculated via local and nonlocal EMT was plotted versus propagation constant (vertical axis) for the considered core layer. As we can see in Figure 5a,b, the influence of propagation constant on effective permittivity tensor components is not significant within the range of propagation constants obtained via solution of characteristic Equations (5)–(8), i.e., $1.1\times 10^{7}m^{-1}<\beta <2.6\times 0^{7}m^{-1}$. It explains the fact that by accounting for nonlocality, it is possible to obtain only a small correction to the propagation properties (compare Figure 4c,d).

#### 3.2.2. Case 2—The Core Layer with 80 nm Dielectric Layer

**Figure 6:**
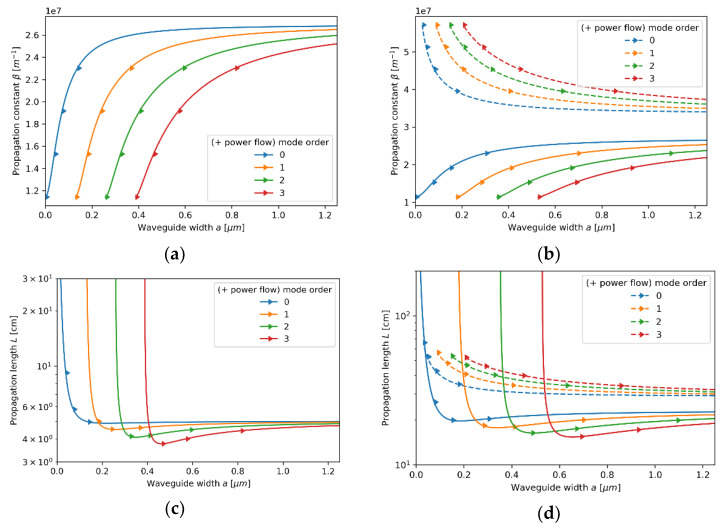
Propagation constant (**a**,**b**) and propagation length (**c**,**d**) of TE modes plotted versus waveguide width for an HMM waveguide with Nb2O5 layer of *t*_Nb2O5_ = 80 nm thickness embedded in the unit cell and described with local (**a**,**c**) and nonlocal EMT (**b**,**d**).

Now, let us consider the propagation properties of TE modes in the waveguide with a core layer based on an 80 nm dielectric layer (see Figure 6a–d). As in the previous case, the local description still predicts propagation properties similar to a conventional dielectric waveguide (compare Figure 3a and Figure 6a). The dielectric character of propagation is also preserved for the nonlocal modes of propagation constants $\beta <2.8\times 10^{7}m^{-1}$ (further regarded as low-*β* modes and depicted with solid lines); only a small change in propagation length can be noted. However, by accounting for the nonlocality, it is possible to predict the existence of an additional branch of modes characterized with higher values of propagation constant $\beta >3.4\times 10^{7}m^{-1}$ (further regarded as high-*β* modes and depicted with dashed lines). Thus, nonlocal description predicts degeneracy of modes, i.e., for a given waveguide width, two TE modes of the same order, but different propagation constants are supported. Moreover, the character of propagation of TE high-*β* modes are substantially divergent from modes of a conventional dielectric waveguide, i.e., decreasing waveguide width enables propagation of modes with higher propagation constant, which implicates higher modal confinement [43]. Additionally, high-*β* modes reveal higher propagation length with increasing order (see Figure 6d). It is noteworthy that a propagation gap, i.e., a range of propagation constants for which only purely imaginary solutions of Equations (5) and (6) are possible, for waves of propagation constant within range $2.7\times 10^{7}\;m^{-1}<\beta <3.4\times 10^{7}{\;m}^{-1}$ can be observed. Again, both local and nonlocal description predict power flow co-directed with propagation direction for each supported mode.

**Figure 7:**
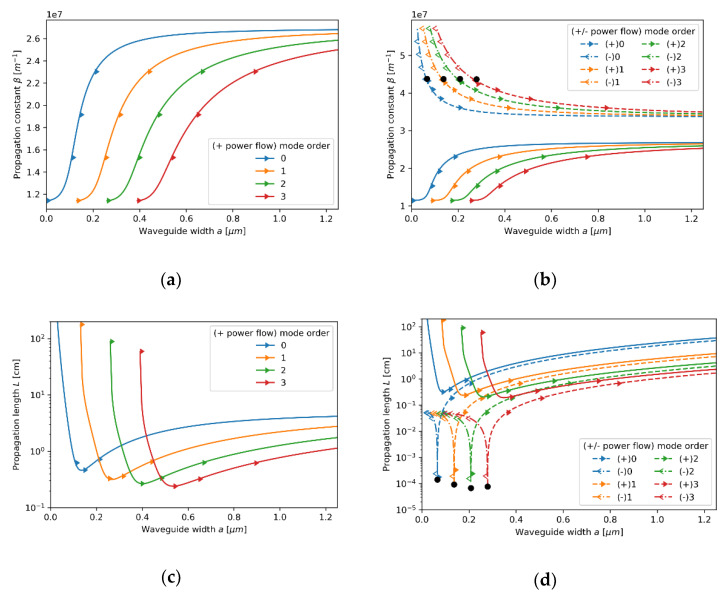
Propagation constant (**a**,**b**) and propagation length (**c**,**d**) of TM modes plotted versus waveguide width for an HMM waveguide with Nb2O5 layer of *t*_Nb2O5_ = 80 nm embedded in the unit cell and described with local (**a**,**c**) and nonlocal EMT (**b**,**d**).

**Figure 8:**
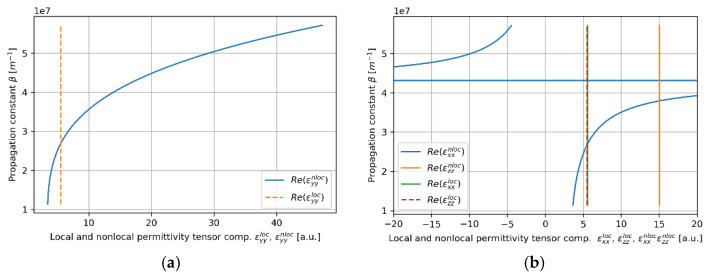
Real parts of components of effective local and nonlocal tensors (horizontal axis) of the core layer with dielectric layer thickness *t*_Nb2O5_ = 80 nm plotted versus propagation constant (vertical axis), which are encountered by TE (**a**) and TM waveguide modes (**b**).

Similarly to TE modes, TM modes with propagation constant within range $2.7\times 10^{7}m^{-1}<\beta <3.4\times 10^{7}m^{-1}$ are not supported in the considered waveguide, i.e., only purely imaginary solutions of Equations (7) and (8) are possible (see Figure 7a–d). For propagation constants $\beta <2.7\times 10^{7}m^{-1}$ (*low-β* modes), the character of modal propagation is very similar to propagation described with the local approach (compare Figure 7a,b). What is more, the degeneracy of modes can be observed, i.e., the existence of an additional modal branch for propagation constant larger than $\beta >3.4\times 10^{7}m^{-1}$ (*high-β* modes) not predicted within the local approach, can be noted (compare Figure 7a,b). It is worth underlining that the existence of an additional branch of TM modes in a nonlocal metamaterial waveguide has been already reported [24]. However, in our case, each *high-β* TM mode reveals a characteristic point (denoted with black dots), i.e., value of propagation constant for which the direction power flow is reversed. Again, the antiparallel and parallel power flow with respect to propagation direction of the given mode is denoted with left- and right-aimed arrows in Figure 7b,d, respectively. The inversion of power flow direction is directly related to the transition between elliptic ($\varepsilon_{xx}^{eff}>0,\varepsilon_{zz}^{eff}>0$) and type II hyperbolic ($\varepsilon_{xx}^{eff}<0,\varepsilon_{zz}^{eff}>0$) dispersion that has been predicted with the help of the nonlocal approach for propagation constant $\beta \approx 4.35\times 10^{7}m^{-1}$ (see Figure 8b). It is worth noting that the modes with propagation constant corresponding to characteristic points reveal power flow tending to zero, i.e., $P_{flow}=0$. This effect is a result of the balance between negative power flow in the core layer and positive power flow in the cladding. It can be also observed that the characteristic points also coincide with minimal values of propagation length (see Figure 7d), which is connected to a strong peak of absorption arising from the transition between elliptic and Type II hyperbolic dispersion [46,47]. Due to that, it can be assumed that modes of propagation constant corresponding to the characteristics point $\beta \approx 4.35\times 10^{7}m^{-1}$ are evanescent, i.e., mode stopping occurs.

#### 3.2.3. Case 3—The Core Layer with 110 Nm Dielectric Layer

**Figure 9:**
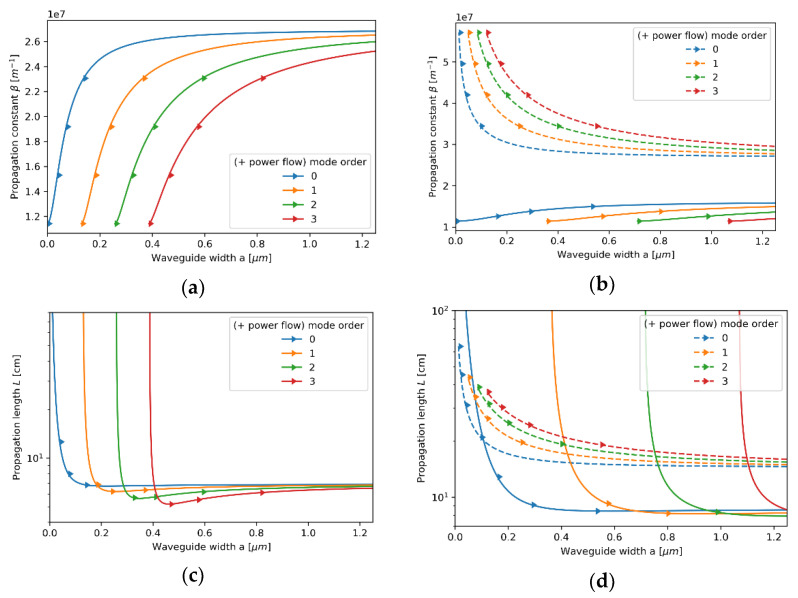
Propagation constant (**a**,**b**) and propagation length (**c**,**d**) of TE modes plotted versus waveguide width for an HMM waveguide with Nb2O5 layer of *t*_Nb2O5_ = 110 nm embedded in the unit cell and described with local (**a**,**c**) and nonlocal EMT (**b**,**d**).

Let us now consider propagation properties of the waveguide with the HMM core layer with the unit cell based on a 110 nm dielectric layer. As we can see in Figure 9a–d, TE modes in the considered waveguide behave similarly as in the previous case. Again, *low-* and *high-β* modes may be observed (see Figure 9a). However, in this case the *low-β* modes are characterized with lower propagation constants in comparison with the local approximation, which is caused by high optical density of the guiding layer (value of $\varepsilon_{yy}^{eff}$) predicted with the help of nonlocal EMT (see Figure 11a). Moreover, the propagation gap, i.e., range of propagation constants for which only purely imaginary solutions of Equations (5) and (6) are possible, is shifted towards lower values of propagation constant, i.e., $1.6\times 10^{7}m^{-1}<\beta <2.9\times 10^{7}m^{-1}$. Moreover, we can observe higher modal confinement in the core layer, which is proportional to the value of the real part of the propagation constant [44], and smaller propagation length in comparison to the previously considered waveguide (compare Figure 6d and Figure 9d).

**Figure 10:**
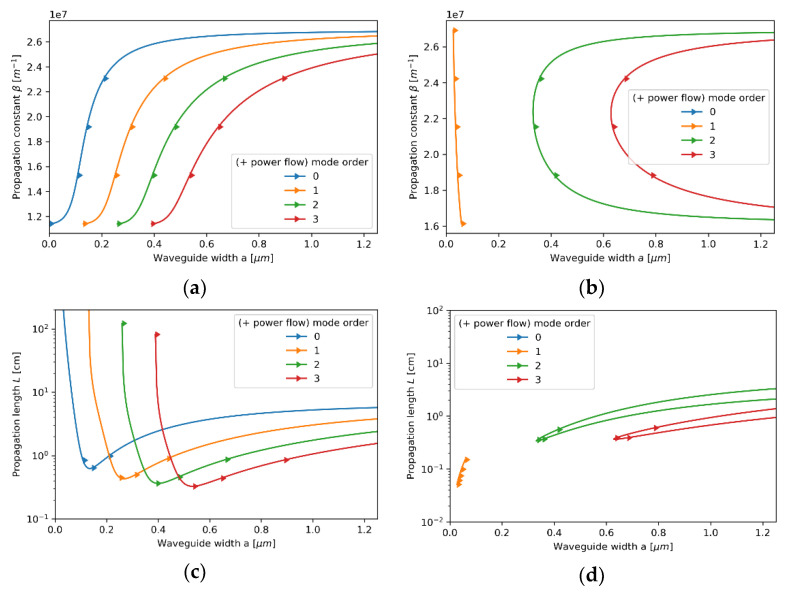
Propagation constant (**a**,**b**) and propagation length (**c**,**d**) of TM modes plotted versus waveguide width for an HMM waveguide with Nb2O5 layer of *t*_Nb2O5_ = 110 nm embedded in the unit cell and described with local (**a**,**c**) and nonlocal EMT (**b**,**d**).

In the case of TM modes, the propagation properties are highly diversified depending on the mode order (see Figure 10a–d). In particular, we can observe that the propagation of the fundamental mode (*m* = 0) is not supported for any waveguide width, while the mode of the first order *m* = 1 may propagate only for a short distance in very thin waveguides (see Figure 10b).

**Figure 11:**
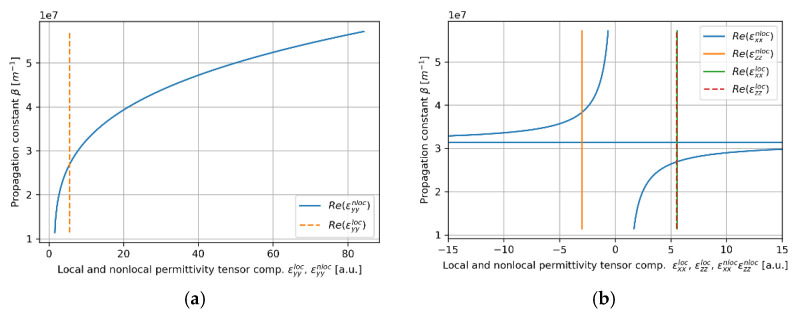
Real parts of components of effective local and nonlocal tensors (horizontal axis) of the core layer with dielectric layer thickness *t*_Nb2O5_ = 110 nm plotted versus propagation constant (vertical axis), which are encountered by TE (**a**) and TM waveguide modes (**b**).

Moreover, modes of higher order *m* > 1 reveal non-zero cut-off widths and modal degeneration, i.e., for a given waveguide width, two modes of different propagation constants are supported in the considered waveguide. It is worth noting that the modal propagation is supported only within a narrow range of propagation constant $1.6\times 10^{7}<\beta <2.7\times 10^{7}m^{-1}$, for which the guiding medium reveals Type I hyperbolic dispersion ($\varepsilon_{xx}^{eff}>0,\varepsilon_{zz}^{eff}<0$) (see Figure 11b). For propagation constants higher than $\beta >3.15\cdot 10^{7}m^{-1}$, the propagation is not supported due to the metallic character ($\varepsilon_{xx}^{eff}<0,\varepsilon_{zz}^{eff}<0$) of the guiding HMM medium.

#### 3.2.4. Case 4—The Core Layer with 175 Nm Dielectric Layer

**Figure 12:**
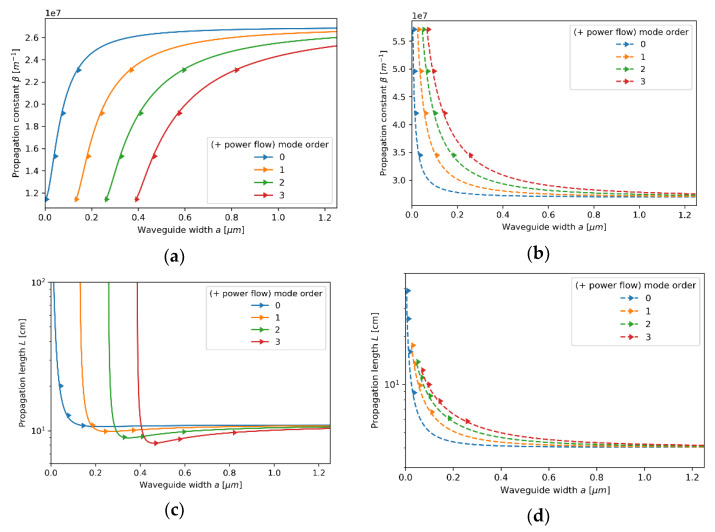
Propagation constant (**a**,**b**) and propagation length (**c**,**d**) of TE modes plotted versus waveguide width for an HMM waveguide with Nb2O5 layer of *t*_Nb2O5_ = 175 nm embedded in the unit cell and described with local (**a**,**c**) and nonlocal EMT (**b**,**d**).

Lastly, we consider the waveguide with the HMM core layer based on a 175nm dielectric layer. In this case, TE modes of the propagation constant $\beta <2.15\cdot 10^{7}m^{-1}$, i.e., low-β modes, are not supported for any waveguide width due to metallic dispersion predicted by nonlocal description ($\varepsilon_{yy}^{eff}<0$) (see Figure 12b. Thus, only high-β modes revealing plasmonic character are allowed for propagation, which substantially differ from the propagation properties predicted with the local approximation (compare Figure 12a,b). Moreover, it can be observed that the propagation length of high-β modes may be considered as long (approx. tens of cm) for the given modal confinement [43,48].

**Figure 13:**
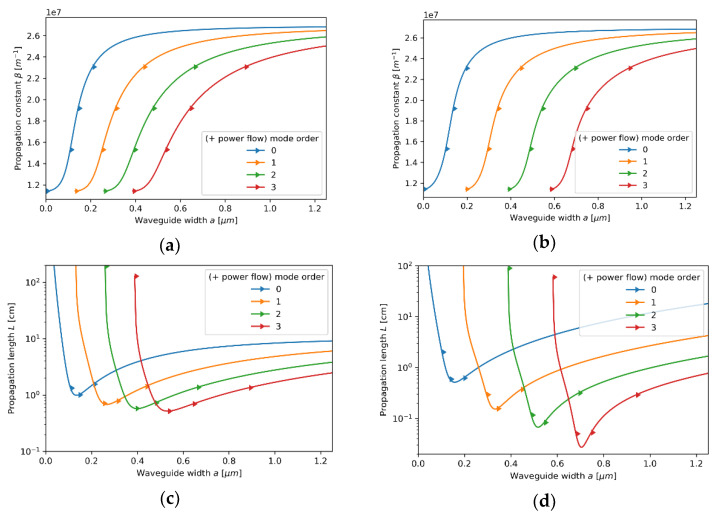
Propagation constant (**a**,**b**) and propagation length (**c**,**d**) of TM modes plotted versus waveguide width for an HMM waveguide with Nb2O5 layer of *t*_Nb2O5_ = 175 nm embedded in the unit cell and described with local (**a**,**c**) and nonlocal EMT (**b**,**d**).

**Figure 14:**
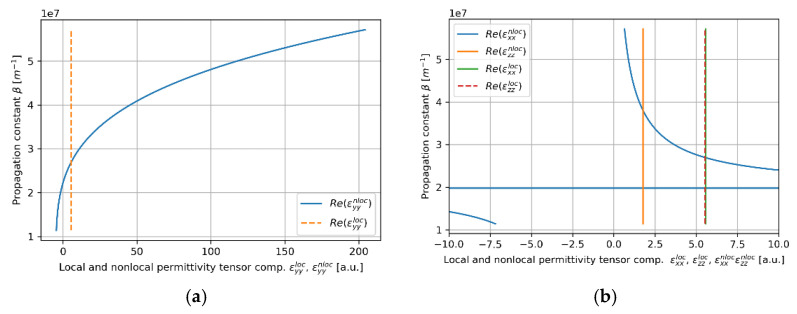
Real parts of components of effective local and nonlocal tensors (horizontal axis) of the core layer with dielectric layer thickness *t*_Nb2O5_ = 175nm plotted versus propagation constant (vertical axis), which are encountered by TE (**a**) and TM waveguide modes (**b**).

Despite the occurrence of Type II hyperbolic dispersion ($\varepsilon_{xx}^{eff}<0,\varepsilon_{zz}^{eff}<0$) for modes of propagation constants $\beta <2\times 10^{7}m^{-1}$, conditions for TM modes in the considered waveguide strongly resemble propagation in the dielectric waveguide (see Figure 14b and compare Figure 13a,b). The only observable difference between the local and nonlocal modes can be noted in the occurrence of cut-off widths, i.e., waveguide widths below which propagation is not supported (compare Figure 13a,b). This phenomenon is caused by low confinement of guided modes leading to the dominant contribution of cladding guiding. What is more, the strong influence of cladding guidance also results in a positive sign of power flow, which means that power flow is parallel to the propagation direction. All of these propagation features may be connected with nonlocal dispersion properties. In particular, existence of only high-β TE modes is a result of metallic dispersion for lower propagation constants $\beta <2.15\times 10^{7}m^{-1}$. Moreover, the transition between elliptic and Type II dispersion and the corresponding absorption peak can be observed in clear minima of propagation length for each TM mode order (see Figure 13d and Figure 14b).

## 4. Conclusions

In this work, we have studied the influence of spatial dispersion on propagation properties of guided modes in a waveguide based on an HMM structure. In the course of our analysis, we have demonstrated for the first time that by changing the geometry of the unit cell, it is possible to control the strength of spatial dispersion and employ it to unveil new effects, such as plasmonic-like modes characterized with long distance propagation, power flow inversion, propagation gap, and mode degeneration (existence of additional branch of modes), which are not otherwise observable within the considered spectral range. Moreover, for the first time, we have reported unusual characteristic points appearing for TM modes, for which power flow tends to zero and propagation is evanescent, i.e., mode stopping occurs. It is worth underlining that this phenomenon has not yet been demonstrated and may serve as a new mechanism for stopping light in a metamaterial waveguide. To summarize, the obtained results address the current problems of designing systems based on nanostructural metamaterials and searching for new possible phenomena. We believe that the presented results will further foster the knowledge of electromagnetic effects arising in metamaterial-based components and systems.

## Data Availability

All reported data and tools are available upon request.
